# Genetic and antigenic characterization of H1 influenza viruses from United States swine from 2008

**DOI:** 10.1099/vir.0.027557-0

**Published:** 2011-04

**Authors:** Alessio Lorusso, Amy L. Vincent, Michelle L. Harland, David Alt, Darrell O. Bayles, Sabrina L. Swenson, Marie R. Gramer, Colin A. Russell, Derek J. Smith, Kelly M. Lager, Nicola S. Lewis

**Affiliations:** 1 Virus and Prion Disease Research Unit, USDA/ARS/NADC, Ames, IA, USA; 2 USDA/APHIS/NVSL, Ames, IA, USA; 3 University of Minnesota, St Paul, MN, USA; 4 Department of Zoology, University of Cambridge, Downing Street, Cambridge CB2 3EJ, UK

## Abstract

Prior to the introduction of the 2009 pandemic H1N1 virus from humans into pigs, four phylogenetic clusters (*α*-, *β*-, *γ*- and *δ*) of the haemagglutinin (HA) gene from H1 influenza viruses could be found in US swine. Information regarding the antigenic relatedness of the H1 viruses was lacking due to the dynamic and variable nature of swine lineage H1. We characterized 12 H1 isolates from 2008 by using 454 genome-sequencing technology and phylogenetic analysis of all eight gene segments and by serological cross-reactivity in the haemagglutination inhibition (HI) assay. Genetic diversity was demonstrated in all gene segments, but most notably in the HA gene. The gene segments from the 2009 pandemic H1N1 formed clusters separate from North American swine lineage viruses, suggesting progenitors of the pandemic virus were not present in US pigs immediately prior to 2009. Serological cross-reactivity paired with antigenic cartography demonstrated that the viruses in the different phylogenetic clusters are also antigenically divergent.

## INTRODUCTION

Influenza in swine is an acute respiratory disease caused by influenza A virus. Influenza A virus contains a negative-sense ssRNA genome organized into eight individual segments, allowing for reassortment and production of novel viruses (Lamb & Krug, 2007). Based upon the major differences within the haemagglutinin (HA) and neuraminidase (NA) proteins, 16 HA and 9 NA subtypes, naturally paired in different combinations, have been identified thus far (Fouchier *et al.*, 2005; Röhm *et al.*, 1996; Webster *et al.*, 1992). HA and NA proteins are encoded by segments 4 and 6 of the viral genome, located on the virion surface, and are the primary target for the host immune response (Skehel & Wiley, 2000). The HA protein is the most important determinant of virulence and host specificity as its binding site and binding pocket recognize sialic acid-containing cell surface receptors on host epithelial cells (Ayora-Talavera *et al.*, 2009; de Wit *et al.*, 2010; Nicholls *et al.*, 2008; Shinya *et al.*, 2006). The remaining six segments encode the following structural and accessory proteins: PB2 (segment 1), PB1 (segment 2), PA (segment 3), NP (segment 5), M1 and M2 (segment 7), NS1 and NS2/NEP (segment 8) (Lamb & Krug, 2007). An additional accessory protein PB1-F2 (Chen *et al.*, 2001) can be encoded by segment 2 as well as the recently identified protein product N40 (Wise *et al.*, 2009), of which little is known. Accessory proteins may confer virulence properties to viruses that express them (McAuley *et al.*, 2007); e.g. PB1-F2 associates with mitochondrial proteins, inducing apoptosis in immune cells (Chen *et al.*, 2001; Zamarin *et al.*, 2006), and NS1 abrogates the expression of antiviral genes in host cells (García-Sastre *et al.*, 1998; Geiss *et al.*, 2002; Lipatov *et al.*, 2005; Seo *et al.*, 2004). Virulence markers as well as factors contributing to inter-species transmission were indentified in the PB2 protein (Gao *et al.*, 2009; Hatta *et al.*, 2001; Mehle & Doudna, 2009; Salomon *et al.*, 2006; Steel *et al.*, 2009; Subbarao *et al.*,1993; Tarendeau *et al.*, 2008; Yamada *et al.*, 2010; Zhu *et al.*, 2010), whereas antiviral-resistance determinants were found in the NA (Le *et al.*, 2005) and M proteins (Marozin *et al.*, 2002).

Classical H1N1 swine viruses (cH1N1), derived from the 1918 pandemic H1N1, were the unique subtype responsible for infection of US swine (Easterday & van Reeth, 1999) until the introduction of a novel H3N2 virus around 1998 changed the status quo of influenza epidemiology in US swine (Vincent *et al.*, 2008). The H3N2 viruses quickly became endemic and then reassorted with extant cH1N1 influenza viruses circulating in North American swine. The H3N2 viruses were demonstrated to have HA, NA and PB1 genes from viruses originating in humans; PA and PB2 genes from viruses originating in avians; and the remaining internal genes, NP, M and NS, of viruses originating in swine, thus giving rise to the triple reassortant designation (Zhou *et al.*, 1999). The human lineage PB1, avian lineages PB2 and PA and swine lineages NP, M and NS found in contemporary influenza viruses of swine are referred to as the triple reassortant internal gene (TRIG) constellation (Vincent *et al.*, 2008). Reassortant viruses have become endemic and co-circulate in most major swine-producing regions of the USA and Canada, including further drift variants of multiple lineages of H3N2, H1N2 and H1N1 (Vincent *et al.*, 2008). Additionally, introduction of H1 in the TRIG backbone viruses with the HA gene of human virus origin (hu-like H1) that are genetically and antigenically distinct from the classical swine H1 lineage were reported (Vincent *et al.*, 2009a). Therefore, in order to best represent the evolution of the currently circulating H1 viruses, a cluster classification has been proposed (Vincent *et al.*, 2009a). Viruses from the cH1N1 lineage evolved over time to form *α*-, *β*- and *γ*-clusters based on the genetic makeup of the HA gene, whereas H1 subtype strains with HA genes most similar to human seasonal H1 viruses form the *δ*-cluster. All four HA cluster gene types can be found with NA genes of either the N1 or N2 subtype.

The concern for the role of pigs in the evolution of influenza A viruses was underscored after the outbreak in humans of the novel 2009 pandemic H1N1. The 2009 pandemic H1N1 has been demonstrated to contain six gene segments of the North American triple reassortant swine lineage with the M and NA from the Eurasian lineage H1N1 (Dawood *et al.*, 2009; Garten *et al.*, 2009). Thus, further investigation regarding the genetic and antigenic evolution of North American influenza virus isolates from swine prior to the emergence of 2009 pandemic H1N1 was warranted. Until recently, however, there were no established tools for the quantitative analysis of antigenic data.

In the present study, we characterized 12 2008 field isolates from swine of the H1 subtype influenza virus in two parallel approaches. The first was based on the full genome sequence and phylogenetic analysis of the eight viral gene segments. The genome sequence analysis was carried out by adopting the next generation 454 genome sequencing technology. The second approach used serological cross-reactivity in the haemagglutination inhibition (HI) assay and antigenic cartography. To our knowledge, this study offers the first antigenic mapping of H1 viruses currently circulating in the swine population.

## RESULTS

### Pyrosequencing technology

The pyrosequencing technology was successfully applied to multiplexed *de novo* sequencing of up to five viruses per region by use of oligo adaptors containing multiplex identifier (MID). Because of the large number of non-influenza virus sequence reads in preliminary sequencing efforts (background RNA from culture source, i.e. MDCK cells) a two-step sequential sucrose semi-purification was performed in the final sequencing run. Additionally, a quantitative real-time RT-PCR for the matrix gene (Spackman & Suarez, 2008) was utilized to quantify influenza virus-specific RNA prior to cDNA synthesis, and real-time PCR for the same matrix gene target was used to semi-quantify viral cDNA just prior to sequencing. The average percentage of the 12 viral genomes sequenced by the Genome Sequencer was 79 % of approximately 13 500 bases. In most cases, large contigs were assembled by the Newbler software for each gene segment for each virus. Comparison of the large *de novo* contigs by blast with known influenza sequences allowed lineage identification for all genes. Sanger sequencing using targeted oligo primers was utilized to walk across gaps or clean up sequences. No large contigs were produced for gene segment 8 of A/swine/Ohio/02026/2008, which was subsequently amplified by standard RT-PCR procedures for sequencing.

### Genetic characterization

We analysed the virus sequences to elucidate the molecular characteristics in those gene segments previously shown to carry virulence factors. All 2008 H1 swine viruses carrying the avian-lineage PB2, contained an E at position 627, a D residue at position 701 and an R residue at position 591. By sequence analysis of the human-lineage PB1 all viral isolates, with the exception of one, contain the full-length coding sequence for the accessory PB1-F2 product. A/swine/Kentucky/02086/2008 had premature stop codons in the PB1-F2 coding sequence after nine residues as in other published PB1 sequences from extant swine viruses (data not shown). All strains contained an N at position 66 of the PB1-F2 mature product. S66 was reported to be associated with increased virulence in the 1918 H1N1 virus and some highly pathogenic H5N1 avian influenza viruses (Conenello *et al.*, 2007).

**Fig. 1.**
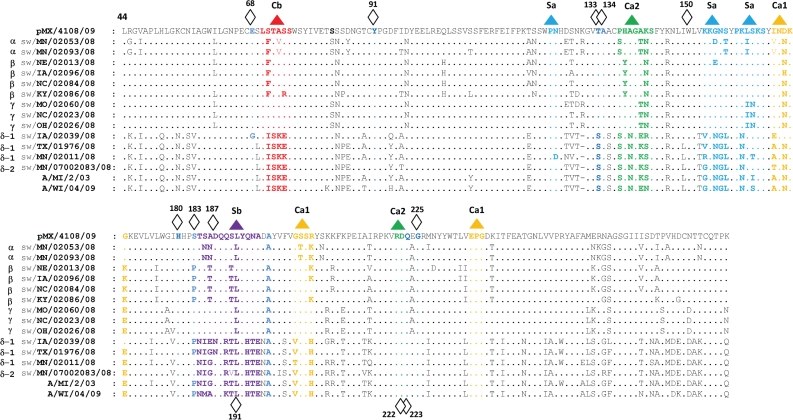
HA1 protein sequence alignment reveals critical amino acid changes between *α*-, *β*- *γ*- and *δ*-clusters. The 12 2008 H1 predicted HA1 proteins were aligned and compared to that of 2009 pandemic H1N1 (A/MX/4108/2009, GenBank accession no. GQ162170) and A/swine/Minnesota/07002083/2007 (*δ*2 cluster, GenBank accession no. FJ611898) and numbered using the mature HA1. Two human seasonal influenza viruses HA sequences were included: A/Michigan/2/2003 (GenBank accession no. CY016324) and A/Wisconsin/04/2009 (GenBank accession no. GQ475852). Amino acid differences are visible in the antigenic sites Cb (red), Ca2 (green), Sa (light blue), Sb (violet) and Ca1 (yellow) between the *α*-, *β*-, *γ*-cluster viruses and the human-like *δ*-cluster viruses. The residues forming the receptor-binding pocket are indicated by a diamond (⋄). Viruses belonging to the *δ*-cluster preserve remnants of the human lineage. A/swine/Iowa/02039/2008 contains a G (blue) at position 68 and N (violet in the Sb antigenic site) at position 187. The 187N human virus-derived residue is present in A/swine/Texas/01976/2008 as well.

Comparison of the HA protein sequences was performed. The 12 2008 H1 isolates contain residues typical of the swine lineage in the receptor-binding pocket with some exceptions: A/swine/Iowa/02039/2008 (*δ*1 HA cluster) showed an exclusively human lineage G residue at position 68 (according to H1 numbering of the mature peptide with 1 being the first amino acid after the signal sequence); A/swine/Iowa/02039/2008 and A/swine/Texas/01976/2008 (*δ*1 HA cluster) display 187N residue as in contemporary H1N1/H1N2 human and swine viruses; *δ*1 and *δ*2 viruses differ in position 133 with respect to the other clusters showing a human-lineage S residue. Moreover, the presence of 187D (or N) suggests a ‘human’ Neu5Ac*α*2-6Gal receptor binding preference (Matrosovich *et al.*, 2000; Neumann & Kawaoka, 2006). Residues of the receptor-binding pocket and variability in the antigenic epitopes are represented in Fig. 1.

The substitutions H274Y and N294S in the NA protein of N1 viruses have been described to confer resistance to the activity of oseltamivir (Le *et al.*, 2005). All the 2008 H1 swine viruses carrying the N1 gene contained the oseltamivir-sensitive genotype. Residue 31S of the M2 protein was previously shown to confer resistance to antiviral molecules amantadine/rimantadine if mutated to an N residue in European swine H1N1, H1N2 and H3N2 influenza viruses (Marozin *et al.*, 2002). The influenza isolates from US swine analysed here displayed the amantadine-sensitive genotype.

The NS1 genes of the 2008 H1 strains do not possess the deletion at position 80–84 or an E at position 92 previously shown to act as virulence factors (Long *et al.*, 2008; Seo *et al.*, 2002). The 2009 pandemic H1N1 was previously shown to lack CPSF30-binding activity (Hale *et al.*, 2010), and all of the 2008 NS1 protein sequences from swine viruses carry the same 108R, 125E and 189G residues responsible for loss of binding, with the exception of a 189S residue present in A/swine/Missouri/02060/2008.

### Phylogenetic analysis

**Fig. 2.**
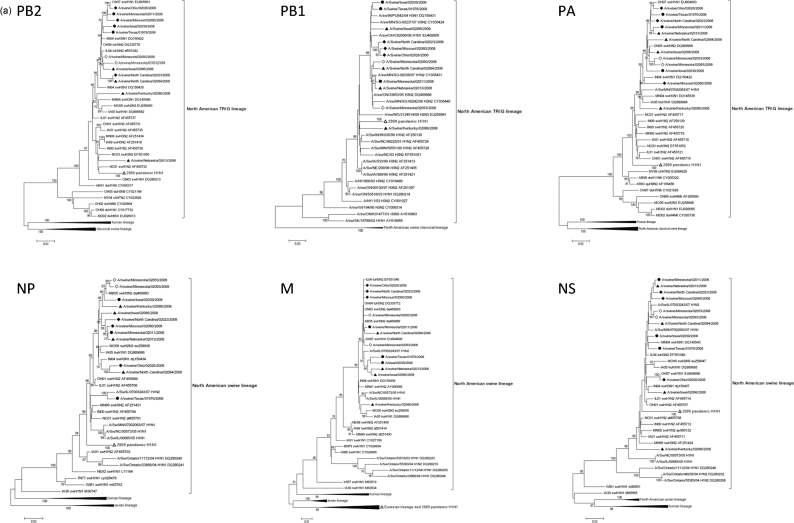

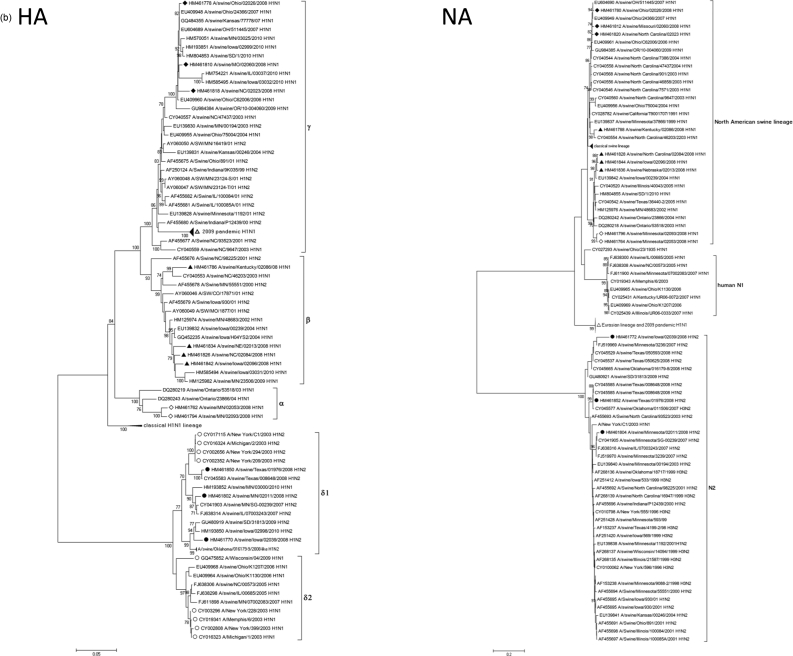
(a, b) Full phylogenetic analysis of the 2008 H1 North American isolates. Neighbour-joining trees inferred from multiple nucleotide sequence alignment of (a), PB2 (2280 nt), PB1 (2274 nt), PA (2151 nt), NP (1497 nt), M (982 nt), NS (844 nt) and (b), HA (1701 nt) and NA (1410 nt) gene segments. HA and NA gene sequences of 24 swine and human pandemic H1N1 (including the five isolates included in the antigenic mapping), five classical H1N1 lineage and 15 *δ*1 isolates were included, but condensed in the HA and NA phylogenetic analyses. Fig. 2(b) HA analysis shows five clusters of related viruses, H1*α*, H1*β*, H1*γ*, H1*δ* and *δ*2 as indicated by the bars on the right of the tree. Bars indicate the estimated numbers of nucleotide substitutions per site. The TRIG constellation is indicated by the bar to the right and other lineages are indicated by group labels of compressed branches. The swine HA-cluster specificity of each 2008 virus is indicated in each tree: open diamond, *α* cluster; closed triangle, *β* cluster; closed diamond, *γ* cluster; closed circle, *δ* cluster; open circle, human seasonal H1N1/N2 influenza viruses; open triangle, 2009 pandemic H1N1. Hu, Human; sw, swine; du, duck; ch, chicken.

The phylogenetic analysis is shown in Fig. 2(a, b). The 12 selected H1 swine influenza viruses represent each of the four previously described HA phylogenetic clusters found in swine and all have the North American TRIG constellation (Fig. 2a). In addition, the HA from the *δ*-cluster viruses were shown to have most likely emerged from two separate introductions of human seasonal HA of an H1N2 and an H1N1 (Vincent *et al.*, 2009a) and can be differentiated by two distinct subclusters, *δ*1 and *δ*2, respectively (Fig. 2b). Variation was demonstrated in the six genes that make up the TRIG, but no HA cluster-specific patterns were detected among the TRIG genes. Viruses belonging to the *δ*-cluster were shown to be paired either with an N1 or an N2 gene consistently of human lineage. Importantly, none of the eight genes from the 2008 H1 viruses clustered with the genes of the 2009 pandemic H1N1 that emerged in the human population in the spring of 2009. In all of the phylogenetic analyses of each gene segment, the pandemic H1N1 formed a distinct and independent branch from the US swine lineage genes.

### Antigenic characterization

**Fig. 3.**
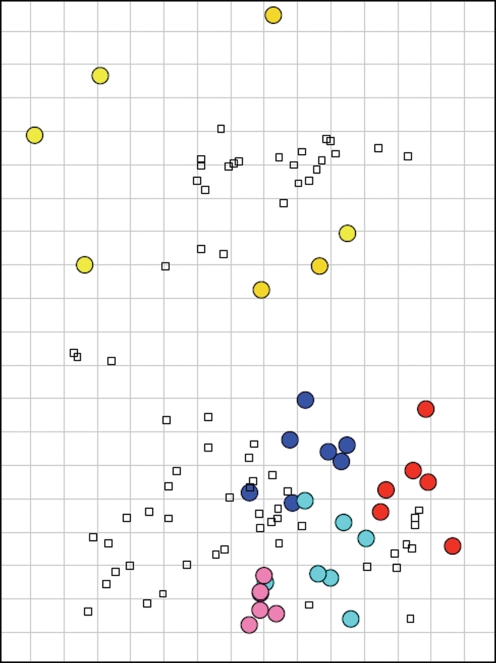
Antigenic map of North American swine influenza viruses subtype H1. The relative positions of strains (coloured dots) and swine hyperimmune antisera (open squares) were computed such that the distances between strains and antisera in the map with the least error represent the corresponding HI measurements (Smith *et al.*, 2004). Strain colour indicates *α*-cluster (cyan), *β*-cluster (pink), *γ*-cluster (blue), *δ*-cluster (gold) and 2009 H1N1 (red). Each grid square represents 1 U of antigenic distance, corresponding to a twofold difference in the HI assay.

Serological cross-reactivity in the HI assay is summarized in Supplementary Tables S1(a, b), available in JGV Online, and the fold-reduction between homologous and heterologous serum and virus pairs is described in Supplementary Tables S1(a, b). Viruses representing the phylogenetic clusters evolved from the cH1N1 swine-lineage demonstrated moderate to strong cross-reactivity within a cluster, especially within recent *β*- and *γ*-cluster viruses. However, cross-reactivity between clusters was more variable, ranging from no cross-reactivity to strong cross-reactivity. The lack of cross-reactivity was most dramatically demonstrated between the cH1N1 swine-lineage clusters and the *δ*-cluster. Indeed, in four of five putative antigenic sites, the *δ*-cluster viruses contain unique amino acid residues making them dramatically different from the other three clusters (Fig. 1). Additional *δ*-cluster viruses were tested against swine influenza antisera by HI (J. R. Ciacci-Zanella and others, unpublished data) and added to the antigenic cartography to better place the 2008 *δ*-cluster viruses. Limited HI cross-reactivity was demonstrated within the *δ*-cluster overall. Future addition of *δ*-cluster viruses and antisera will improve their placement in the swine H1 antigenic map. The 2008 H1 viruses tended to be mapped antigenically in accordance with their phylogenetic cluster as demonstrated in Fig. 3, and represent at least five antigenically distinct clusters of H1 viruses co-circulating in US swine in 2008.

Antisera generated against two isolates of 2009 pandemic H1N1 were additionally tested against select 2007–2008 swine H1 viruses and results reported in Table 3(a, b) and included in the antigenic cartography are depicted in Fig. 3. Decreased fold-differences in cross-reactivity were evident between the 2009 pandemic H1N1 antisera and *α*- and *β*-cluster influenza isolates from US swine. Moderate cross-reactivity was demonstrated between the 2009 antisera and *γ*-cluster swine isolates tested (Table 3a, b). No cross-reactivity was found between 2009 pandemic H1N1 antisera and viruses belonging to the *δ*-cluster (data not shown). Cross-reactivity between swine influenza antisera and 2009 pandemic H1N1 viruses was shown in a recent study (Vincent *et al.*, 2010). Three 2009 pandemic H1N1 isolated from swine (Supplementary Table S3, available in JGV Online) were tested against the 2008 serum panel and included in the antigenic cartography analysis. Human and swine 2009 pandemic H1N1 isolates mapped together in the antigenic cartography (Fig. 3).

## DISCUSSION

The purpose of this study was to characterize the genetic and antigenic properties of 12 representative 2008 H1 influenza viruses isolated from US swine. The viruses were collected the year prior to the emergence and global spread of the 2009 pandemic H1N1 virus and represent the predominant viruses circulating in US swine at that time. The 454 genome sequencing technology described in this report was successfully applied to multiplexed *de novo* sequencing for nearly all segments of each virus. Large contigs were generated that allowed identification of the sequences as influenza A virus as well as lineage determination. Recent reports using oligo enrichment (Ramakrishnan *et al.*, 2009) or amplicon (Höper *et al.*, 2009) based techniques for 454 sequencing likely improves coverage of the full influenza A virus genome compared with the completely *de novo* method used here. Advancement in next generation genome sequencing continues to evolve as this relatively new technology is applied to viral RNA genomes.

Genetic diversity was demonstrated in all gene segments, but most notably in the HA gene with five distinct genetic clusters (*α*-, *β*-, *γ*-, *δ*1 and *δ*2). All *α*-, *β*- and *γ*-cluster H1 viruses studied were paired with an N1 subtype of the North American swine lineage. The three *δ*1 were paired with an N2 subtype. In contrast to HA, no patterns of clustering according to H1 cluster could be extended to the genes composing the TRIG. This suggests that reassortment and drift of the H1 HA is occurring randomly on the TRIG backbone. None of the *δ*-cluster sequences used in the phylogenetic analysis from our virus set or published sequences were paired with a swine-lineage N1 as found for viruses in the *α*-, *β*- and *γ*-clusters. Thus, the human-lineage *δ*-cluster HA shows a unique preference for the human-lineage N1 and N2. The human N2 gene was introduced into pigs nearly 10 years ago (Vincent *et al.*, 2008) and is now well-established in the swine population. The human-lineage N2 from the H3N2 viruses are closely related to the NA genes of the seasonal human H1N2 from which the *δ*1 viruses evolved. An optimal ‘HA/NA pairing’ in association with the TRIG could be advantageous for the virus in terms of transmission and replication properties as confirmed by recent experiments in pigs (Ma *et al.*, 2010).

The influenza virus polymerase plays a critical role in the adaptation of avian viruses to mammalians. Generally, aa 627 in the PB2 protein is almost exclusively K in human influenza isolates and E in avian influenza isolates (Subbarao *et al.*, 1993). Additionally, the mutation E627K was found to be responsible for host range, tissue tropism and increased virulence of avian viruses in mammals (Fouchier *et al.*, 2004; Gao *et al.*, 2009). In addition, a D701N amino acid change has been found to confer high pathogenicity to an H5N1 in experimentally infected mice (Li *et al.*, 2005). Mammalian-type residues K627 and N701 are found in some H5N1 or H7N7 (Fouchier *et al.*, 2004) and are implicated in expanding the transmission range in ferrets (Van Hoeven *et al.*, 2009) and guinea pigs (Steel *et al.*, 2009). The 2009 pandemic H1N1 possess E627 and an R591 that has been recently recognized to sustain efficient replication in mammalian cells, compensating for the lack of K627 (Mehle & Doudna, 2009; Yamada *et al.*, 2010). Of the publicly available swine TRIG PB2 sequences, approximately 30 % contain R591 (Mehle & Doudna, 2009). Accordingly, the entire panel of the 2008 TRIG H1 isolates reported in this study contained PB2-R591, suggesting that this genotype may be selected in the swine host. Additional PB2 sequence analysis and evaluation *in vivo* in swine is warranted.

Antigenic cartography is a theory and associated computational method that resolves the paradoxes in the interpretation of antigenic data and makes possible high-resolution quantitative analyses and visualization of binding assay data (de Jong *et al.*, 2007; Garten *et al.*, 2009; Huang *et al.*, 2009; Park *et al.*, 2009; Russell *et al.*, 2008; Smith *et al.*, 2004). By using antigenic cartography and phylogenetic analysis, we showed that five antigenic (*α*-, *β*-, *γ*-clusters, *δ*1- and *δ*2-subclusters) of H1 influenza A viruses, co-circulate in the US pig population, but have limited HI cross-reactivity between them. Antigenic cross-reactivity was limited between H1 clusters and supports the practice of combining multiple H1 viruses with H3N2 viruses in polyvalent vaccines used to control influenza in US swine population as well as many other regions around the world (http://www.aphis.usda.gov/animal_health/vet_biologics/publications/CurrentProdCodeBook.pdf). Established influenza viruses undergo antigenic drift in the swine host, allowing survival and selection of certain viral clusters in the host population. The H1 viruses will likely continue to mutate and evolve in the swine host as a consequence of evolutionary and immunological pressures, allowing evasion of the immune system of the host or only partially protective immunity. Further study is required to evaluate the usefulness of antigenic mapping for vaccine strain selection through experimental efficacy studies in pigs.

Our previous experiment (Vincent *et al.*, 2010) demonstrated cross-reactivity using 2009 H1N1 as antigen and sera from pigs immunized with *γ*-cluster swine viruses from 2007 and 2008. The *γ*-cluster is the clade with which the HA from 2009 H1N1 is most closely related (Figs 2b and 3). Thus, prior exposure to some H1 swine subtypes is likely to provide some level of protection against infection with the 2009 human pandemic variant. This is also suggested by data from human epidemiological studies that showed high prevalence of neutralizing antibodies against 2009 pandemic H1N1 in people born before 1930 (Itoh *et al.*, 2009; Munster *et al.*, 2009). Moreover, immunization in mice with human H1N1 viruses that circulated before 1945 (e.g. specific antibodies against 1918 H1N1 or related viruses) is sufficient for immune protection from the 2009 pandemic H1N1 (Manicassamy *et al.*, 2010).

The 2009 pandemic H1N1 underscores the potential risk to the human population from additional influenza virus subtypes and genotypes with the swine influenza TRIG backbone and demonstrates the potential for viruses with genes from swine lineages to emerge and cause human illness. However, all eight gene segments from the 2009 pandemic H1N1 formed clusters separate from US swine lineage viruses, suggesting neither the pandemic H1N1 nor closely related progenitor viral genes were present in influenza viruses from US swine prior to 2009.

Recent epidemiological data (M. R. Gramer and others, unpublished data) suggest that the number of swine influenza outbreaks in which *δ*-cluster viruses were recognized as causative agents increased in recent years (up to 40 % of isolated H1 swine influenza viruses in 2009 and early 2010), thus quickly becoming a dominant genotype in the USA. An expanded antigenic map with additional contemporary *δ*-cluster isolates and further investigation of their antigenic relationship with seasonal human viruses are therefore warranted.

The presence of typical ‘human-like’ residues in the receptor-binding pocket of the HA in two *δ*-cluster strains isolated from pigs demonstrates that swine viruses may efficiently preserve human-adapted phenotypes. The preservation of human-like residues in the swine host may allow potential novel reassortant influenza viruses to spill back into the human population, and this may be particularly important to consider for the *δ*-cluster swine viruses. Increased surveillance and monitoring by sequence and antigenic analysis of enzootic influenza viruses of swine as well as the 2009 pandemic H1N1 in the swine population worldwide are critical for understanding the dynamic ecology of influenza A viruses in this susceptible host population. Likewise, development of a vaccine strain selection system through surveillance and antigenic characterization of contemporary viruses is critical for controlling swine influenza viruses and reducing the risk of such reassortment events with the current 2009 pandemic H1N1 or other emerging viruses in the future.

## METHODS

### Virus selection.

Twelve H1N1 or H1N2 viruses representing each of the genetic clusters of subtype H1 from 2008 were received from the University of Minnesota Veterinary Diagnostic Laboratory (UMVDL) for use in this study. The viruses were isolated from outbreaks of respiratory disease in pigs from routine diagnostic cases submitted to the UMVDL. Viruses isolated in 2008 from the USA and Canada were selected from a database of 375 viral RNA sequences generated from diagnostic cases, each 900 nt long, of the H1 subtype (Table 1). The sequences were stored in a group database [Los Alamos National Laboratory, Influenza Sequence Database (http://www.flu.lanl.gov/)] and phylogenetic analyses were performed based on sequence alignment by the clustal w method using the megalign program from the Lasergene package (dnastar). From the phylogenetic analyses of the HA1 region of the HA gene, 12 viruses were selected for inclusion in this study to represent each of the H1 clusters of influenza viruses from North American swine circulating in 2008. Viruses were grown in Madin−Darby canine kidney (MDCK) cells in culture. Inactivated viruses for immunization were prepared at approximately 128 HA units per 50 μl (or maximum HA titre) with inactivation by UV irradiation and addition of a commercial adjuvant (Emulsigen D; MVP Laboratories) at a ratio of 4 : 1 (v/v) virus to adjuvant. Virus isolates contained in the inactivated vaccines and selected for full genome analysis are reported in Table 2. Similarly prepared immune sera from additional studies conducted at USDA NADC (Vincent *et al.*, 2006, 2009a) were included in HI assays and antigenic mapping and are described in Table 2.

### 454 genome sequencing technology.

The 12 selected viruses from clinical cases were grown at low passage number (*P*<5) in MDCK cell culture, concentrated and semi-purified on a 0.5 M sucrose cushion. Virus pellets were resuspended overnight at 4 °C in 0.5 ml STE buffer (10 mM NaCl, 10 mM Tris, pH 8.0 and 1 mM EDTA, pH 8.0). Resuspended virus samples were treated with 10 U RNase-free DNase I (Promega) and incubated for 10 min at room temperature, then treated with 500 U RNase inhibitor (RNase-In; Ambion) and 5 μl of 0.1 M dithiothreitol (Invitrogen). An equal volume of 2× LES buffer (0.2 M LiCl, 10 mM EDTA and 2.0 % SDS) was added to each sample, treated with 200 μg proteinase K (Invitrogen) and incubated for 30 min at 56 °C. Nucleic acid was extracted with phenol/chloroform and resuspended in 50 μl 10 mM Tris/HCl (pH 7.5) followed by library preparation for *de novo* pyrosequencing in a Genome Sequencer FLX system (454 Life Sciences) with the manufacturer's recommendations and reagents. Briefly, extracted viral RNA was fragmented, reverse transcribed and ligated to oligonucleotide adaptors containing MID labels. The MID-labelled viral cDNA samples were pooled and used to prepare sequencing beads via Roche's GS-FLX standard chemistry emulsion-based PCR. Each pool contained viral cDNA from three to five different viruses. Prepared beads were loaded onto 16 regions on a GS-FLX standard chemistry pico-titre plate according to manufacturer's recommendations and sequenced using the GS-FLX LR 70 standard chemistry. Sequencing reads were compared to an influenza database created from >85 000 influenza sequences extracted from GenBank in December, 2008. Using the blast results, influenza-specific sequencing reads were extracted and subsequently assembled with the Roche GS *de novo* Assembler (Newbler) version 2.0 software. Gene segments with large gaps were closed by traditional primer based sequencing using an ABI 3100 (Applied Biosystem) genetic analyser. The sequence contigs were analysed using SeqMan (dnastar). The 2008 H1 sequences generated were deposited into GenBank with the accession numbers proceeding from segment 1 to segment 8 as presented in Table 2. Human and swine influenza virus sequences adopted for the phylogenetic analysis were retrieved from the Influenza Virus Sequence Database (http://www.ncbi.nlm.nih.gov/genomes/FLU/Database/nph-select.cgi?go=database). For the HA and NA analyses, sequences recruitment was restricted to those exhibiting a minimum of 1000 nt. Sequence alignment for each individual gene segment and phylogenetic analyses were conducted using mega version 4 and the evolutionary distances were computed using the maximum composite likelihood method. Statistical support was provided by bootstrapping over 1000 replicates and bootstrap values >70 are indicated at the corresponding node (Tamura *et al.*, 2007).

### Animals, serological assay and antigenic cartography.

Four-week-old cross-bred pigs were obtained from a herd free of both influenza virus and porcine reproductive and respiratory syndrome virus (PRRSV) infections. Animals were housed, treated and screened for anti-influenza antibodies as described previously (Vincent *et al.*, 2009b). Two pigs per virus were immunized with inactivated virus combined with commercial adjuvant by an intramuscular route. Two doses of vaccine were given approximately 2–3 weeks apart; pigs with HI titres <1 : 80 after the second dose were given a third dose of vaccine prior to final blood collection. At the end of the vaccination period, pigs were humanely euthanized with pentobarbital (Sleepaway; Fort Dodge Animal Health, Fort Dodge, IA) approximately 2 weeks after the final vaccination for blood collection.

Sera were heat inactivated at 56 °C and treated as described elsewhere (Vincent *et al.*, 2010) and the HI assays were performed with turkey red blood cells with standard techniques (WHO Manual on Animal Influenza Diagnosis and Surveillance). The reciprocal of the ratio between heterologous and homologous HI titres for individual serum samples was calculated to indicate the fold-change between heterologous and homologous reactions.

The quantitative analyses of the antigenic properties of 2008 H1 viruses were performed using antigenic cartography. In an antigenic map, the distance between antiserum point S and antigen point A corresponds to the difference between the log_2_ of the maximum titre observed for antiserum S against any antigen and the log_2_ of the titre for antiserum S against antigen A. Therefore, each titre in an HI table can be thought of as specifying a target distance for the points in an antigenic map. Modified multidimensional scaling methods are then used to arrange the antigen and antiserum points in an antigenic map to best satisfy all the target distances specified by the HI data. The result is a map in which the distance between points best represents antigenic distance as measured by the HI assay. Because antisera are tested against multiple antigens, and antigens tested against multiple antisera, many measurements are used to determine the position of the antigen and antiserum points in an antigenic map, thus potentially increasing the accuracy of point placement beyond that of individual HI measurements.

## Supplementary Material

Supplementary Tables

## Figures and Tables

**Table 1.** 2008 UMVDL H1 swine influenza virus sequence dataset from which the 12 2008 H1 viruses were selected

**Pig location**	***α*-Cluster**	***β*-Cluster**	***γ*-Cluster**	***δ*-Cluster**	**Total**
**USA**					
Iowa	0	11	6	14	**31**
Illinois	3	6	26	8	**43**
Indiana	0	0	1	0	**1**
Kansas	1	4	2	4	**11**
Kentucky	0	5	0	0	**5**
Michigan	0	0	1	1	**2**
Minnesota	6	58	16	48	**128**
Missouri	6	13	7	3	**29**
North Carolina	0	2	30	18	**50**
Nebraska	0	7	1	2	**10**
Ohio	0	0	11	5	**16**
Oklahoma	0	5	0	16	**21**
South Dakota	1	0	2	1	**4**
Texas	0	2	1	7	**10**
Wisconsin	0	3	5	1	**9**
**Canada**					
Manitoba	4	0	0	0	**4**
Saskatchewan	1	0	0	0	**1**
**Total**	**22**	**116**	**109**	**128**	**375**

**Table 2.** 2008 H1 swine influenza viruses (left column) included in the genetic and antigenic study and previous H1 swine influenza virus isolates used for the generation of antisera Sequence GenBank accession numbers proceeding from segment gene 1 to segment gene 8 for each 2008 H1 isolate. Sw, Swine; p, pandemic.

**2008 H1**	**GenBank accession nos**	**Cluster**	**Subtype**	**NADC H1**	**Cluster**	**Subtype**
A/SW/MN/02053/2008	HM461759–HM461766	*α*	H1N1	A/SW/MN/37866/1999	cH1	H1N1
A/SW/MN/02093/2008	HM461791–HM461798	*α*	H1N1	A/SW/IA/1973	cH1	H1N1
A/SW/IA/02096/2008	HM461839–HM461846	*β*	H1N1	A/SW/WI/1/1968	cH1	H1N1
A/SW/KY/02086/2008	HM461783–HM461790	*β*	H1N1	A/SW/IA/1945	cH1	H1N1
A/SW/NE/02013/2008	HM461831–HM461838	*β*	H1N1	A/SW/IA/15/1930	cH1	H1N1
A/SW/NC/02084/2008	HM461823–HM461830	*β*	H1N1	A/SW/IA/00239/2004	*β*	H1N1
A/SW/NC/02023/2008	HM461815–HM461822	*γ*	H1N1	A/SW/NC/36883/2002	*β*	H1N1
A/SW/OH/02026/2008	HM461775–HM461782	*γ*	H1N1	A/SW/OH/511445/2007	*γ*	H1N1
A/SW/MO/02060/2008	HM461807–HM461814	*γ*	H1N1	A/SW/KS/00246/2004	*γ*	H1N2
A/SW/TX/01976/2008	HM461847–HM461854	*δ*1	H1N2	A/SW/MN/00194/2003	*γ*	H1N2
A/SW/IA/02039/2008	HM461767–HM461774	*δ*1	H1N2	A/SW/MN/1192/2001	*γ*	H1N2
A/SW/MN/02011/2008	HM461799–HM461806	*δ*1	H1N2	A/SW/MN/07002083/2007	*δ*2	H1N1
				A/SW/NC/00573/2005	*δ*2	H1N1
				A/SW/IL/00685/2005	*δ*2	H1N1
				A/SW/IL07003243/2007	*δ*1	H1N2
				A/MX/4108/2009	2009p	H1N1
				A/CA/04/2009	2009p	H1N1
				A/SW/IL/5265/2009	2009p	H1N1
				A/SW/IL/32974/2009	2009p	H1N1
				A/SW/MN/8761/2010	2009p	H1N1

**Table 3.** (a, b) HI titres for individual swine serum samples (a), fold-reduction compared with homologous HI titre (b) Reciprocal HI titres for individual serum samples against human isolates of 2009 pandemic H1N1 are reported for 2007–2008 US H1 swine influenza viruses representing the swine-lineage *α*-, *β*-, and *γ*-clusters. p, Pandemic; phylogenetic clusters are indicated by the corresponding letter in front of the virus or antiserum names.

**Viruses**	**Sera**			
	**p#960A**	**p#962A**	**p#963A**	**p#966A**
**(a)**				
p A/CA/04/09	640*	640*	320	320
p A/MX/4108/09	640	640	640*	1280*
*α* A/Sw/MN/02053/08	10	40	20	20
*α* A/Sw/MN/02093/08	160	160	40	40
*β* A/Sw/IA/02096/08	80	80	80	40
*β* A/Sw/KY/02086/08	80	160	160	80
*β* A/Sw/NE/02013/08	40	20	80	20
*β* A/Sw/NC/02084/08	80	160	80	80
*γ* A/Sw/NC/02023/08	160	160	160	160
*γ* A/Sw/OH/02026/08	80	80	80	80
*γ* A/Sw/MO/02060/08	80	80	80	40
*γ* A/Sw/OH/511445/07	80	80	80	80
**(b)**				
p A/CA/04/09	*	*	2	2
p A/MX/4108/09	1	1	*	*
*α* A/Sw/MN/02053/08	64	16	32	32
*α* A/Sw/MN/02093/08	4	4	16	16
*β* A/Sw/IA/02096/08	8	8	8	16
*β* A/Sw/KY/02086/08	8	4	4	8
*β* A/Sw/NE/02013/08	16	32	8	32
*β* A/Sw/NC/02084/08	8	4	8	8
*γ* A/Sw/NC/02023/08	4	4	4	4
*γ* A/Sw/OH/02026/08	8	8	8	8
*γ* A/Sw/MO/02060/08	8	8	8	16
*γ* A/Sw/OH/511445/07	8	8	8	8

*Homologous antiserum and virus reactions.
