# Supplementary material for: Genetic and antigenic characterization of H1 influenza viruses from United States swine from 2008
Source: J Gen Virol. 2011 Apr;92(Pt 4):919–30. doi: 10.1099/vir.0.027557-0 (PMC3133703; doi:10.1099/vir.0.027557-0)
Supplement: Supplementary Tables [file supp_92_4_919__index.html]

Supplementary Tables 

# Genetic and antigenic characterization of H1 influenza viruses from United States swine from 2008

## 

### Genetic and antigenic characterization of H1 influenza viruses from United States swine from 2008, by A. Lorusso, A. L. Vincent, M. L. Harland, D. Alt, D. O. Bayles, S. L. Swenson, M. R. Gramer, C. A. Russell, D. J. Smith, K. M. Lager and N. S. Lewis

*Journal of General Virology* vol. **92**, part 4, pp. 919 - 930

**Supplementary Table S1a.** Reciprocal HI titres for individual serum samples are reported for 24 different US swine influenza viruses.

**Supplementary Table S1b.** HI titres for individual serum samples.

**Supplementary Table S2a.** The ratio between homologous and heterologous reciprocal HI titres for individual serum samples are reported for 24 different US H1 swine influenza viruses.

**Supplementary Table S2a.**Fold-reduction compared to homologous HI titre.

**Supplementary Table S3.** Reciprocal HI titres for individual serum samples are reported for three 2009 pandemic H1N1 isolates.   
  
[Single PDF file]  (118 KB)
