# Supplementary material for: Genetic and antigenic characterization of H1 influenza viruses from United States swine from 2008
Source: J Gen Virol. 2011 Apr;92(Pt 4):919–30. doi: 10.1099/vir.0.027557-0 (PMC3133703; doi:10.1099/vir.0.027557-0)
Supplement: Supplementary Tables [file supp_92.4.919_Supplementary_tables.pdf]

Supplementary Table S1a. (a, b) Reciprocal HI titres for individual serum samples are reported for 24 different US swine influenza viruses.

Tables S1a and S1b were split due to size constraints. Phylogenetic clusters are colour-coded by α-cluster (cyan), β-cluster (pink), γ-cluster (blue) and δ-cluster (yellow). Shaded cells indicate homologous antiserum and virus and double bar boxes indicate reactions within phylogenetic clusters.

.

SERA →	874	875	876	877	532	595	589	590	596	870	871	872	873	878	879	880	881	577	558
VIRUS ↓																			
A/SW/MN/02053/2008	320	320	160	160	320	320	160	640	320	20	80	40	40	40	40	<10	20	160	80
A/SW/MN/02093/2008	160	320	160	320	320	320	80	2560	160	10	10	<10	20	10	10	<10	20	80	80
A/SW/MN/37866/1999	20	40	<10	40	160	160	320	640	320	<10	<10	<10	40	20	10	<10	10	80	40
A/SW/IA/1973	20	40	20	20	320	5120	1280	1280	1280	10	20	10	80	20	20	<10	40	160	80
A/SW/IA/1945	10	20	<10	20	160	160	1280	320	1280	<10	<10	<10	20	<10	<10	<10	<10	80	<10
A/SW/WI/1/1968	40	80	20	80	320	160	320	640	640	10	20	20	80	20	20	10	20	160	80
A/SW/IA/15/1930	20	20	<10	20	80	320	640	640	640	<10	<10	10	20	20	10	<10	<10	40	20
A/SW/IA/02096/2008	160	40	40	40	160	640	80	1280	640	80	320	80	160	160	80	<10	40	640	640
A/SW/KY/02086/2008	160	40	80	80	640	80	20	640	640	10	20	80	640	80	80	80	20	160	160
A/SW/NE/02013/2008	80	40	20	20	160	160	80	640	640	40	80	40	160	640	160	80	40	320	160
A/SW/NC/02084/2008	160	40	40	20	320	160	160	640	640	40	80	40	160	320	160	160	80	320	320
A/SW/IA/00239/2004	80	40	40	80	320	320	320	640	320	40	40	40	160	160	160	80	40	640	160
A/SW/NC/36883/2002	20	20	40	40	160	320	160	640	320	40	40	40	80	80	40	40	40	320	160
A/SW/NC/02023/2008	20	40	10	20	160	80	80	320	40	<10	<10	<10	10	<10	10	<10	<10	80	20
A/SW/OH/02026/2008	40	40	80	40	320	320	160	320	320	10	40	80	80	40	40	<10	40	160	1280
A/SW/MO/02060/2008	20	40	10	20	160	160	80	160	<10	<10	<10	<10	<10	<10	<10	<10	<10	40	<10
A/SW/OH/511445/2007	80	320	160	160	640	320	320	2560	320	80	160	40	160	160	160	80	80	320	160
A/SW/KS/00246/2004	80	160	40	80	320	160	80	320	80	<10	<10	<10	20	10	40	<10	20	80	40
A/SW/MN/00194/2003	40	40	10	10	80	20	20	40	20	20	10	10	10	20	10	<10	<10	20	10
A/SW/MN/1192/2001	40	40	10	10	320	80	160	80	<10	10	10	<10	20	10	10	<10	10	80	<10

Lorusso, A., Vincent, A. L., Harland, M. L., Alt, D., Bayles, D. O., Swenson, S. L., Gramer, M. R., Russell, C. A., Smith, D. J., Lager, K. M. and Lewis, N. S. (2011). Genetic and antigenic characterization of H1 influenza viruses from United States swine from 2008. *J Gen Virol* **92**, 919–930.

A/SW/TX/01976/2008	<10	<10	<10	<10	<10	<10	<10	<10	<10	<10	<10	<10	<10	<10	<10	<10	<10	<10	<10
A/SW/IA/02039/2008	<10	<10	<10	<10	<10	<10	<10	<10	<10	<10	<10	<10	<10	<10	<10	<10	<10	<10	<10
A/SW/MN/02011/2008	<10	<10	<10	<10	20	<10	<10	<10	<10	<10	<10	<10	<10	<10	<10	<10	<10	<10	<10
A/SW/MN/07002083/2007	<10	<10	<10	<10	<10	<10	<10	40	<10	<10	<10	<10	<10	<10	<10	<10	<10	<10	<10

Supplementary Table S1b. HI titres for individual serum samples.

SERA →	864	865	866	867	868	869	821	581	560	592	882	883	884	885	886	887	328
VIRUS ↓																	
A/SW/MN/02053/2008	40	40	20	640	1280	640	320	320	40	80	<10	<10	<10	<10	<10	<10	<10
A/SW/MN/02093/2008	80	160	40	2560	1280	1280	1280	640	40	160	<10	<10	<10	<10	<10	<10	<10
A/SW/MN/37866/1999	80	80	10	1280	640	640	320	80	20	80	<10	<10	<10	<10	<10	<10	<10
A/SW/IA/1973	40	80	<10	640	640	320	320	160	<10	1280	<10	<10	<10	<10	<10	<10	<10
A/SW/IA/1945	40	40	<10	1280	640	640	160	80	<10	40	<10	<10	<10	<10	<10	<10	<10
A/SW/WI/1/1968	<10	40	10	1280	1280	1280	320	80	20	80	<10	<10	<10	<10	<10	<10	<10
A/SW/IA/15/1930	<10	20	<10	640	320	160	80	20	<10	40	<10	<10	<10	<10	<10	<10	<10
A/SW/IA/02096/2008	40	80	20	320	640	160	320	320	20	160	<10	<10	<10	<10	<10	<10	<10
A/SW/KY/02086/2008	40	40	<10	320	160	80	160	80	20	1280	<10	<10	<10	<10	<10	<10	<10
A/SW/NE/02013/2008	20	80	10	640	640	160	160	40	<10	80	<10	<10	<10	<10	<10	<10	<10
A/SW/NC/02084/2008	40	80	10	1280	640	320	320	160	20	320	<10	<10	<10	<10	<10	<10	<10
A/SW/IA/00239/2004	160	80	80	640	640	640	160	160	40	320	<10	<10	<10	<10	<10	<10	<10
A/SW/NC/36883/2002	40	40	20	320	640	320	80	40	40	20	<10	<10	<10	<10	<10	<10	<10
A/SW/NC/02023/2008	320	320	40	640	640	640	640	320	80	80	<10	<10	<10	<10	<10	<10	<10
A/SW/OH/02026/2008	160	640	80	1280	640	640	640	640	640	320	<10	<10	<10	<10	<10	<10	10
A/SW/MO/02060/2008	80	320	40	1280	1280	2560	640	160	40	40	<10	<10	<10	<10	<10	<10	<10
A/SW/OH/511445/2007	320	1280	160	2560	2560	1280	2560	1280	320	320	80	80	20	20	320	80	<10
A/SW/KS/00246/2004	160	160	160	640	1280	640	640	640	160	320	40	20	20	80	20	20	<10
A/SW/MN/00194/2003	40	80	40	320	320	320	160	80	640	80	40	20	20	40	40	40	10
A/SW/MN/1192/2001	160	640	40	1280	640	1280	640	320	80	640	10	<10	<10	10	<10	<10	<10
A/SW/TX/01976/2008	<10	<10	<10	<10	<10	<10	<10	<10	<10	<10	40	40	<10	<10	<10	<10	10
A/SW/IA/02039/2008	<10	<10	<10	<10	<10	<10	<10	<10	<10	<10	10	40	320	40	<10	<10	<10

Lorusso, A., Vincent, A. L., Harland, M. L., Alt, D., Bayles, D. O., Swenson, S. L., Gramer, M. R., Russell, C. A., Smith, D. J., Lager, K. M. and Lewis, N. S. (2011). Genetic and antigenic characterization of H1 influenza viruses from United States swine from 2008. *J Gen Virol* **92**, 919–930.

A/SW/MN/02011/2008	<10	<10	<10	<10	<10	<10	<10	<10	20	40	40	40	40	20	2560	2560	40
A/SW/MN/07002083/2007	<10	10	<10	<10	10	10	<10	<10	<10	<10	160	80	20	<10	<10	<10	640

Lorusso, A., Vincent, A. L., Harland, M. L., Alt, D., Bayles, D. O., Swenson, S. L., Gramer, M. R., Russell, C. A., Smith, D. J., Lager, K. M. and Lewis, N. S. (2011). Genetic and antigenic characterization of H1 influenza viruses from United States swine from 2008. *J Gen Virol* **92**, 919–930.

Supplementary Table S2a. (a, b) The ratio between homologous and heterologous reciprocal HI titres for individual serum samples are reported for 24 different US H1 swine influenza viruses.

Tables S2a and S2b were split due to size constraints. Phylogenetic clusters are colour coded by α-cluster (cyan), β-cluster (pink), γ-cluster (blue) and δ-cluster (yellow). Solid cells indicate homologous antiserum reactions and virus and double bar boxes indicate reactions within phylogenetic clusters. An eightfold or greater loss in reactivity is considered a significant reduction that may be predictive of decreased protection from challenge.

SERA →	874	875	876	877	532	595	589	590	596	870	871	872	873	878	879	880	881	577	558
VIRUS ↓																			
A/SW/MN/02053/2008			1	2	0.5	16	8	1	2	4	4	2	16	16	4	>16	4	4	2
A/SW/MN/02093/2008	2	1			0.5	16	16	0.25	4	8	32	>8	32	64	16	>16	4	8	2
A/SW/MN/37866/1999	16	8	>16	8		32	4	1	2	>8	>32	>8	16	32	16	>16	8	8	4
A/SW/IA/1973	16	8	8	16	0.5		1	0.5	0.5	8	16	8	8	32	8	>16	2	4	2
A/SW/IA/1945	32	16	>16	16	1	32		2	0.5	>8	>32	>8	32	>64	>16	>16	>8	8	>16
A/SW/WI/1/1968	8	4	8	4	0.5	32	4		1	8	16	4	8	32	8	16	4	4	2
A/SW/IA/15/1930	16	16	>16	16	2	16	2	1		>8	>32	8	32	32	16	>16	>8	16	8
A/SW/IA/02096/2008	2	8	4	8	1	8	16	0.5	1			1	4	4	2	>16	2	1	0.25
A/SW/KY/02086/2008	2	8	2	4	0.25	64	64	1	1	8	16			8	2	2	4	4	1
A/SW/NE/02013/2008	4	8	8	16	1	32	16	1	1	2	4	2	4			2	2	2	1
A/SW/NC/02084/2008	2	8	4	16	0.5	32	8	1	1	2	4	2	4	2	1			2	0.5
A/SW/IA/00239/2004	4	8	4	4	0.5	16	4	1	2	2	8	2	4	4	1	2	2		1
A/SW/NC/36883/2002	16	16	4	8	1	16	8	1	2	2	8	2	8	8	4	4	2	2	
A/SW/NC/02023/2008	16	8	16	16	1	64	16	2	16	>8	>32	>8	64	>64	16	>16	>8	8	8
A/SW/OH/02026/2008	8	8	2	8	0.5	16	8	2	2	8	8	1	8	16	4	>16	2	4	0.125
A/SW/MO/02060/2008	16	8	16	16	1	32	16	4	>64	>8	>32	>8	>64	>64	>16	>16	>8	16	>16
A/SW/OH/511445/2007	4	1	1	2	0.25	16	4	0.25	2	1	2	2	4	4	1	2	1	2	1
A/SW/KS/00246/2004	4	2	4	4	0.5	32	16	2	8	>8	>32	>8	32	64	4	>16	4	8	4

Lorusso, A., Vincent, A. L., Harland, M. L., Alt, D., Bayles, D. O., Swenson, S. L., Gramer, M. R., Russell, C. A., Smith, D. J., Lager, K. M. and Lewis, N. S. (2011). Genetic and antigenic characterization of H1 influenza viruses from United States swine from 2008. *J Gen Virol* **92**, 919–930.

A/SW/MN/00194/2003	8	8	16	32	2	256	64	16	32	4	32	8	64	32	16	>16	>8	32	16
A/SW/MN/1192/2001	8	8	16	32	0.5	64	8	8	>64	8	32	>8	32	64	16	>16	8	8	>16
A/SW/TX/01976/2008	>16	>16	>16	>32	>16	>512	>128	>64	>64	>8	>32	>8	>64	>64	>16	>16	>8	>64	>16
A/SW/IA/02039/2008	>16	>16	>16	>32	>16	>512	>128	>64	>64	>8	>32	>8	>64	>64	>16	>16	>8	>64	>16
A/SW/MN/02011/2008	>16	>16	>16	>32	8	>512	>128	>64	>64	>8	>32	>8	>64	>64	>16	>16	>8	>64	>16
A/SW/MN/07002083/2007	>16	>16	>16	>32	>16	>512	>128	16	>64	>8	>32	>8	>64	>64	>16	>16	>8	>64	>16

Lorusso, A., Vincent, A. L., Harland, M. L., Alt, D., Bayles, D. O., Swenson, S. L., Gramer, M. R., Russell, C. A., Smith, D. J., Lager, K. M. and Lewis, N. S. (2011). Genetic and antigenic characterization of H1 influenza viruses from United States swine from 2008. *J Gen Virol* **92**, 919–930.

Supplementary Table S2a. Fold-reduction compared to homologous HI titre.

SERA →	864	865	866	867	868	869	821	581	560	592	882	883	884	885	886	887	328
VIRUS ↓																	
A/SW/MN/02053/2008	8	8	4	2	1	4	8	2	16	8	>4	>4	>32	>4	>256	>256	>64
A/SW/MN/02093/2008	4	2	2	0.5	1	2	2	1	16	4	>4	>4	>32	>4	>256	>256	>64
A/SW/MN/37866/1999	4	4	8	1	2	4	8	8	32	8	>4	>4	>32	>4	>256	>256	>64
A/SW/IA/1973	8	4	>8	2	2	8	8	4	>64	0.5	>4	>4	>32	>4	>256	>256	>64
A/SW/IA/1945	8	8	>8	1	2	4	16	8	>64	16	>4	>4	>32	>4	>256	>256	>64
A/SW/WI/1/1968	>32	8	8	1	1	2	8	8	32	8	>4	>4	>32	>4	>256	>256	>64
A/SW/IA/15/1930	>32	16	>8	2	4	16	32	32	>64	16	>4	>4	>32	>4	>256	>256	>64
A/SW/IA/02096/2008	8	4	4	4	2	16	8	2	32	4	>4	>4	>32	>4	>256	>256	>64
A/SW/KY/02086/2008	8	8	>8	4	8	32	16	8	32	0.5	>4	>4	>32	>4	>256	>256	>64
A/SW/NE/02013/2008	16	4	8	2	2	16	16	16	>64	8	>4	>4	>32	>4	>256	>256	>64
A/SW/NC/02084/2008	8	4	8	1	2	8	8	4	32	2	>4	>4	>32	>4	>256	>256	>64
A/SW/IA/00239/2004	2	4	1	2	2	4	16	4	16	2	>4	>4	>32	>4	>256	>256	>64
A/SW/NC/36883/2002	8	8	4	4	2	8	32	16	16	32	>4	>4	>32	>4	>256	>256	>64
A/SW/NC/02023/2008			2	2	2	4	4	2	8	8	>4	>4	>32	>4	>256	>256	>64
A/SW/OH/02026/2008	2	0.5			2	4	4	1	1	2	>4	>4	>32	>4	>256	>256	64
A/SW/MO/02060/2008	4	1	2	1			4	4	16	16	>4	>4	>32	>4	>256	>256	>64
A/SW/OH/511445/2007	1	0.25	0.5	0.5	0.5	2		0.5	2	2	0.5	0.5	16	2	8	32	>64
A/SW/KS/00246/2004	2	2	0.5	2	1	4	4		4	2	1	2	16	0.5	128	128	>64
A/SW/MN/00194/2003	8	4	2	4	4	8	16	8		8	1	2	16	1	64	64	64
A/SW/MN/1192/2001	2	0.5	2	1	2	2	4	2	8		4	>4	>32	4	>256	>256	>64
A/SW/TX/01976/2008	>32	>32	>8	>128	>128	>256	>256	>64	>64	>64			>32	>4	>256	>256	64
A/SW/IA/02039/2008	>32	>32	>8	>128	>128	>256	>256	>64	>64	>64	4	1			>256	>256	>64
A/SW/MN/02011/2008	>32	>32	>8	>128	>128	>256	>256	>64	32	16	1	1	8	2			16
A/SW/MN/07002083/2007	>32	32	>8	>128	128	256	>256	>64	>64	>64	0.25	0.5	16	>4	>256	>256	

Lorusso, A., Vincent, A. L., Harland, M. L., Alt, D., Bayles, D. O., Swenson, S. L., Gramer, M. R., Russell, C. A., Smith, D. J., Lager, K. M. and Lewis, N. S. (2011). Genetic and antigenic characterization of H1 influenza viruses from United States swine from 2008. *J Gen Virol* **92**, 919–930.

Supplementary Table S3. Reciprocal HI titres for individual serum samples are reported for three 2009 pandemic H1N1 isolates.

Phylogenetic clusters are colour-coded by α-cluster (cyan), β-cluster (pink) and γ-cluster (blue). (b) After the serum number indicates a subsequent bleed day with respect to that reported in Table 3(a, b) in the main text. Shaded cells indicate homologous antiserum and virus.

SERA →	960B	962B	963B	966B	874	875	876	877	870	871	873	878	879	880	881	864	865	866	867	868	869
VIRUS ↓																					
A/CA/04/09	5120	5120	1280	1280																	
A/MX/4108/09	5120	5120	1280	1280																	
A/sw/IL/5265/2010	2560	2560	640	1280	<10	<10	<10	<10	<10	<10	<10	<10	<10	<10	<10	40	80	10	10	80	80
A/sw/IL/32974/2009	1280	1280	640	640	20	<10	<10	<10	<10	<10	<10	<10	<10	<10	<10	10	40	10	80	160	160
A/sw/MN/8761/2010	2560	2560	640	640	40	<10	<10	<10	<10	<10	<10	<10	<10	<10	<10	80	160	10	160	320	320

Lorusso, A., Vincent, A. L., Harland, M. L., Alt, D., Bayles, D. O., Swenson, S. L., Gramer, M. R., Russell, C. A., Smith, D. J., Lager, K. M. and Lewis, N. S. (2011). Genetic and antigenic characterization of H1 influenza viruses from United States swine from 2008. *J Gen Virol* **92**, 919–930.
